# Implementing the World Health Organization safe childbirth checklist in a district Hospital in Rwanda: a pre- and post-intervention study

**DOI:** 10.1186/s40748-018-0075-3

**Published:** 2018-04-04

**Authors:** Eugene Tuyishime, Paul H. Park, Dominique Rouleau, Patricia Livingston, Paulin Ruhato Banguti, Rex Wong

**Affiliations:** 1 Department of Anesthesia, Critical Care and Emergency Medicine, University of Rwanda, Kigali, Rwanda; 2 University Teaching Hospital of Kigali, Kigali, Rwanda; 3 Partners In Health - Inshuti Mu Buzima, Rwinkwavu, Kayonza, Rwanda; 4 Brigham and Women’s Hospital, Boston, MA USA; 5 University of Global Health Equity, Kigali, Rwanda; 6 Harvard Medical School, Harvard University, Cambridge, USA; 7 Dalhousie University, Halifax, Canada; 8 King Faisal Hospital, Kigali, Rwanda; 9 Yale Global Health Leadership Institute, Yale University, New Haven, CT USA

**Keywords:** Quality improvement, Essential birth practice, WHO safe childbirth checklist, Evidence-based, Maternal mortality, Child mortality, Rwanda

## Abstract

**Background:**

Worldwide maternal mortality remains high, with approximately 830 maternal deaths occurring each day. About 90% of these deaths occur in low-income countries. Evidenced-based essential birth practices administered during routine obstetrical care and childbirth are key to reducing maternal and neonatal deaths. The WHO Safe Childbirth Checklist (SCC) is a low-cost tool designed to ensure birth attendants perform 29 essential birth practices (EBP) at four critical periods in the birth continuum. This study aimed to evaluate compliance with EBP in Masaka District Hospital both before and after the implementation of the WHO-SCC.

**Methods:**

This quality improvement project took place in the Masaka District Hospital in Rwanda. Observations of the 29 EBPs were done before and after WHO SCC implementation. The implementation process consisted of providing training in the use of the checklist to all clinical staff and posting SCC posters at different locations in the maternity unit.

**Results:**

A total 391 birth events were observed pre-intervention and 389 post-intervention. The overall EBP compliance rate increased from 46% pre-intervention to 56% post-intervention (*P* = 0.005). Significant improvements were seen in 11 out of 29 EBPs.

**Conclusion:**

The implementation of the WHO SCC improved the overall EBP compliance rate in Masaka District Hospital. Determining the root cause of low compliance rate of some EBP may allow for more successful implementation of EBP interventions in the future. After further study, the SCC should be considered for scale up.

## Background

Reducing maternal mortality is a major focus worldwide. It was estimated that about 830 maternal deaths occurred every day around the globe in 2015; with 95% of the deaths having occurred in low-income countries and over 60% in Sub Saharan Africa [1]. In the 2000 UN Millennium Summit, many world leaders collaboratively developed eight Millennium Development Goals (MDG) to reduce extreme poverty globally [2]. By 2015, less than half of the countries had achieved MDG 4 - reduce child mortality, and MDG 5 – improve maternal health [3]. The use of evidenced-based essential birth practices (EBPs) for routine prenatal care and management of complications during childbirth is key to achieving high quality of care and reducing maternal and child deaths [4]. Following the success of the surgical safety checklist at reducing surgical complications [5–8], WHO developed the Safe Childbirth Checklist (SCC) in 2009 [9, 10]. The WHO Safe Childbirth Checklist (SCC) includes 29 essential birth practices targeting major causes of maternal deaths, intrapartum-related stillbirths and neonatal deaths that occur in facilities around the world [1]. This low-cost tool is designed to be accessible to birth attendants to ensure that timely, lifesaving practices are performed for every facility-based birth. The SCC is focused on care delivered for births at term gestation as these represent the overwhelming majority of births. The SCC is designed to address quality of care at four critical periods in the birth continuum: on admission to facility, at the time of pushing (or before cesarean delivery), soon after birth (within 1 h), and at discharge [11]. Previous studies have shown that use of the SCC increases the compliance rate of EBP in low resources settings [12, 13]. The use of the WHO-SCC has also been shown to contribute to the improvement of quality of care through three mechanisms: it serves as a reminder for the essential care practices; it is an advocacy tool for data collection and performance improvement; and it standardizes care so that every patient receives the essential components [13]. A one-center study conducted in India showed an increase in the EBP compliance of 150% after the introduction of SCC [13]. Another study conducted in three hospitals in India showed improvements in 13 EBP [14].

Rwanda has achieved the MDG 4 and MDG 5 and the maternal and neonatal mortality rates have decreased considerably. Between 2000 and 2015 maternal deaths were reduced from 1071 to 210 per 100,000 births, and neonatal death from 42 to 19 per 1000 births ([15]; World Bank, 2015). However, cost effective interventions remain needed in order to reach the Sustainable Development Goals (SDGs) of decreasing the maternal mortality rate to below 70 per 100,000 birth and neonatal mortality rate to below 12 per 1000 births by 2030 [16]. To our knowledge one health facility in Rwanda has implemented the WHO SCC, but its effectiveness for improving EBP compliance had never been evaluated. In response to this need, our study evaluates the effectiveness of implementing the WHO SCC on EBP compliance in one district hospital in Rwanda. The results of this study could inform the scaling up of the SCC in other district hospitals in Rwanda.

## Methods

### Aim

This study aimed to evaluate the effectiveness of implementing the WHO SCC on EBP compliance in one district hospital in Rwanda.

### Setting

The study was conducted in Masaka District Hospital in Rwanda. The hospital has 35 maternity beds and offers both emergency obstetric care and caesarean deliveries. In 2016, the average hospital length of stay was 1 day after vaginal delivery and 3 days after cesarean section. Approximately 500 vaginal deliveries are performed monthly. The maternity unit team includes eight physicians, 20 midwives and three nurses. Despite the large number of deliveries performed, the hospital had not adopted the WHO Safe Childbirth Checklist prior to our intervention.

### Study design

This study utilized a pre- and post-intervention design to evaluate the effectiveness of implementing the WHO SCC checklist on the compliance of essential birth practices.

The pre-intervention between January and February 2017 included a baseline assessment of the compliance on 29 EBP in the maternity service of Masaka District Hospital. Data was collected through observation by the data collection team over 14 consecutive day shifts (7 am – 7 pm) and 4 night shifts (7 pm – 7 am). The data collecting team was comprised of the principal investigator, two midwives and two medical students. After receiving a 2-day training, the data collectors were assigned to observe and record the completion of EBP at the four different “pause points”: 1) at admission (5 items), 2) before pushing and 1 min after delivery (11 items), 3) within 1 h post-deliver (6 items), and 4) within 1 h before discharge (7 items) (Table 1). Since the primary objective of this study was to observe the health care professionals’ behavior toward EBP, not the health outcomes of individual mothers, we conducted four independent cross sectional sampling on different women at the four pause points. The same observations were repeated between March and April 2017 to collect data post-implementation of the WHO SCC. To ensure data quality, the principal investigator performed random observations and data collections 2 days per week and compared the results to those collected by data collectors. Feedback was provided to data collectors when needed.

**Table 1:** Pre- and post-intervention essential birth practice compliance rate

EBP	pre-intervention	post-intervention	*P*-value
Pause point 1. “On admission”			
Observations (n)	106	95	–
1. Appropriate referral	100 (100%)	100 (100%)	1.000
2. Maternal temperature ^**^	75 (70.8%)	51 (53.7%)	0.014^**^
3. Maternal blood pressure	90 (84.9%)	81 (85.3%)	1.000
4. Partograph started	59 (55.7%)	45 (47.4%)	0.26
5. Birth companion ^**^	1 (0.9%)	7 (7.4%)	0.028^**^
Average	65 (61%)	56.8 (60%)	0.848
Pause point 2. “Before pushing and within 1 min after delivery”			
Observations (n)	92	98	
6. Water used to clean hands	22 (22.4%)	18 (18.2%)	0.483
7. Soap used to clean hands	18 (18.4%)	17 (17.2%)	0.854
8. Alcohol rub used	2 (2%)	3 (3%)	1.000
9. Clean towel ^**^	63 (64.3%)	84 (85.7%)	0.001^**^
10. Clean gloves used	100(100%)	100(100%)	1.000
11. Clean scissors/ blade	88 (89.8%)	84 (84.8%)	0.392
12. Clean cord ligature/tie ^**^	58 (59.2%)	89 (90.8%)	< 0.001^**^
13. Aspiration bulb or Mucus extractor	66 (67.3%)	70 (70.7%)	0.646
14. Clean pads for mother ^**^	43 (43.9%)	61 (61.6%)	0.015^**^
15. Oxytocin	76 (77.6%)	84 (84.8%)	0.206
16. Birth companion present ^**^	0 (0%)	10 (10.2%)	0.002^**^
Average	48.7 (53%)	56.4 (58%)	0.591
Pause point 3. “Within 1 h after delivery”			
Observation (n)	92	98	
17. Newborn weight taken^**^	76 (77.6%)	94 (94.9%)	< 0.001^**^
18. Newborn temperature	5 (5.1%)	4 (4.1%)	1.000
19. Neonatal bag and mask prepared	69 (70.4%)	69 (69.7%)	1.000
20. Baby placed skin-to-skin^**^	48 (49.0%)	89 (89.9%)	< 0.001^**^
21. baby still skin-to-skin at 1 h	2 (2.0%)	6 (6.1%)	0.279
22. Breastfeeding initiated^**^	32 (32.7%)	47 (47.5%)	0.042^**^
Average	38.7 (42%)	51.5 (53%)	0.161
Pause point 4. “Before discharge”			
Observation (n)	101	98	
23. Maternal blood pressure obtained^**^	14 (13.9%)	58 (58.6%)	< 0.001^**^
24. Mother’s temperature ^**^	3 (3.0%)	54 (54.5%)	< 0.001^**^
25. Baby’s temperature	1 (1.0%)	5 (5.1%)	0.115
26. Baby’s respiratory rate	1 (1.0%)	5 (5.1%)	0.115
27. Baby’s feeding	89 (88.1%)	84 (84.8%)	0.54
28. Family planning options discussed	94 (93.1%)	88 (88.9%)	0.333
29. Danger signs explained ^**^	1 (1.0%)	66 (66.7%)	< 0.001^**^
Average ^**^	29 (29%)	51.4 (52%)	0.001^**^
Overall compliance			
Observation (n)	391	389	
Overall average ^**^	46%	56%	0.005^**^

^**^Statistically significant at *P* = 0.05

### Sample

Women who were present at Masaka District Hospital maternity ward during the four pause points were included in the study. Mothers were excluded from the study if they declined or were unable to provide consent. Patients who had seizures, cardiac arrest, severe respiratory distress, hemodynamic instability, severe bleeding, and mental disorders were also excluded from this study.

### Intervention

In February 2017, the principal investigator provided a 2-day training session on the implementation of SCC to all clinical staff and administrators at the maternity unit. The principle investigator also provided clear and open communication with staff so that they understood the importance and objective of this project. Regular feedback was provided to the staff to ensure both timely positive reinforcement and corrective actions. All necessary equipment, included documentations and thermometers, was available for the maternity unit to promote compliance. SCC posters were posted on the walls around the delivery wards as reminders to clinicians of the EBPs and their importance. Staff coaching was also provided during implementation and training.

### Data analysis

The overall compliance rates of EBP at the four pause points, and for each of the 29 EBP items, were compiled and compared before and after the intervention. Chi-Square tests were used for the analyses, using SPSS v.20 with *P*-value set at 0.05.

## Results

All hospital staff involved in childbirth agreed to participate in the study. In total, 590 patients gave their informed consent to participate in the study, 299 before and 291 after the implementation. All patients from pause point II were also followed for pause point III using the same informed consent.

For all four IV pause points, a total of 391 childbirth EBP events were observed pre-intervention and 389 post-intervention.

The overall compliance rate of EBP practices significantly increased from 46% pre-intervention to 56% post-intervention (*p* = 0.005). Three pause points (pause points II, III, and IV) had shown improvement on the compliance of EBP; particularly for pause point IV – before discharge, which significantly increased from 29% pre-intervention to 52% post-intervention (*p* = 0.001). The average compliance rate of EBP during admission (pause point I) showed slight decreased from 61% pre-intervention to 60% post-intervention, although statistically not significant (*p* = 0.848) (Table 1).

Out of the 29 EBPs, 11 (38%) showed statistically significant increase in compliance rates and 1 (3.4%) showed a statistically significant decrease post-intervention. Among the 11 EBPs showing significant improvement, 1 out of 5 was from pause point I, 4 out of 11 from pause point II, 3 out of 6 from pause point III and 3 out of 7 from pause point IV.

The improved EBP from pause point I was 1) birth companion presence on admission (*P* = 0.028); from pause point II were 2) clean towel use (*P* = 0.001), 3) clean cord ligature/tie use (*P* < 0.001), 4) clean pad for the mother use (*P* = 0.015), 5) birth companion presence before pushing and within 1 min after delivery (*P* = 0.002); from pause point III were 6) newborn weight (P < 0.001), 7) breastfeeding initiation (P < 0.001), 8) initiation of baby’s skin to skin at 1 min (*P* = 0.042); and from pause point IV were 9) maternal blood pressure check before discharge (P < 0.001), 10) maternal temperature check before discharge (P < 0.001), and 11) danger signs explanation (P < 0.001) (Table 1). The EBP with significant decrease from pause point I was maternal temperature on admission (*P* = 0.014) (Table 1).

## Discussion

The implementation of the WHO SCC was associated with an overall increase in compliance with EBP by the staff in the maternity ward of Masaka District Hospital, suggesting the feasibility of using the SCC to potentially improve maternal and perinatal care. Our result was consistent with other previous studies although the magnitude of improvement of our study was not as substantial as in other studies [13, 14]. The study conducted in India showed the EBP compliance rate increase as high as 150% after the introduction of SCC over a period of 3 months evaluation [13]. In contrast, our study post-intervention evaluation was only 3 weeks, much shorter than the other study. Such longer evaluation period also may have allowed the involved health care professionals to enhance their competency and familiarity of the EBP using the checklist. Longer-term follow up evaluation in the future may show more comparable changes. The highest increase in EBP was at pause point IV (before discharge), which also had the lowest pre-intervention compliance rate (29%). We speculate that the medical needs of the patients who were ready to be discharged may have been considered relatively less urgent, and thus received less attention and prioritization compared to those of patients at earlier stages of their delivery, particularly if there was a shortage of staff. Measuring the blood pressure and temperature of mother, or explaining the danger signs to the family before discharge could easily be missed or conducted but not properly documented. The introduction of SCC reminded the staff to perform these EBP despite their busy workload. Furthermore, the EBPs at this pause point mostly focus on patient education and prevention of complications after discharge; this kind of tasks requires relatively less human resources and equipment, thus should be easier to accomplish once reminded by the SCC.

The only EBP that showed statistical significant decrease from pre-intervention to post-intervention was taking maternal temperature at pause point I. During the implementation period, there was one staff on leave and the workload was distributed to other staff. From our discussion with them, many staff believed taking the temperature upon admission was relatively less important compared to the other EBP practices during active labor. Thus when the workload is high, the seemingly less important practices is more likely to be missed by the staff. However further investigation on this is required.

The keys to the success of the project was the application of many quality improvement principles during training and coaching including strategic problem solving skills, internal ownership, regular monitoring and evaluation, and engagement of leadership [17–19]. The implementation was taken on using a team approach. The team included the researchers, the clinical director, the head of maternity unit, and the head of post-delivery unit, who all worked collaboratively in the preparation, assessment, implementation, and evaluation. The team not only measured the baseline practices but also assessed the working situation to identify and understand the barriers to proper EBP performance. Feedback was provided to the staff regularly to ensure timely positive reinforcement and corrective actions.

The support from the hospital senior management team and the relevant staff was vital. Clear directives from management allowed the staff to understand the importance of this project. Involving the staff in the maternity ward from the planning stage of the project allowed them to take ownership of it and fostered early buy-in. We identified some highly motivated staff who were eager to improve the performance of their department and volunteered to coach during the implementation period. The early and continuous engagement of key actors helped to build a culture of teamwork and promote sustainability. With the support from the hospital administration and quality improvement team, the WHO Safe Childbirth Checklist has become part of the hospital protocol. All new staff working in the maternity unit will receive an orientation to the checklist. Such support from the hospital could increase the sustainability of the continuous use of the WHO SCC despite the end of this study.

Similar to what was described in other studies [20, 21], the implementation of the SCC, including its planning and training activities created an extra workload for the already small staff in the post-delivery unit.

This study has several limitations. Firstly, we could not eliminate Hawthorne effect; we could not control whether or not the staff changed their behavior due to their being observed. However, because the same approach was used both at baseline and post-intervention observations, we believe the Hawthrone effect should be similar in both pre- and post-intervention data collections. Secondly, our study was conducted in only one district hospital and the results and conclusions may not be applicable to other hospital settings. Thirdly, the patient numbers in our studied was small during the relatively short follow-up period (3 weeks) compared to some other similar studies, for example, 3 months evaluation in India [13] despite we detected statistical significance. Also, the clinical improvement was small, however as staff become more familiar with the SCC, the learning effect will probably increase the compliance rate. Longer term follow-up is needed to understand the sustainability and, naturally, larger study will be needed before any scale up of the project. Lastly, we acknowledge that there is a risk of bias in our study design. By excluding women with pregnancy complications, the study design could have potentially inflated the uptake at pause points 2, 3 and 4. After considerable discussion among hospital management team and project team, we have decided when pregnancy complication occurs, all staff should focus on saving lives rather than worrying about observing behavior and collecting data. By not having an observer in the already limited space room could ensure the health care professionals to be more efficient and not worrying about being observed and thus causing extra stress.

## Conclusion

The implementation of the WHO SCC improved the overall EBP compliance rate in Masaka District Hospital. Determining the root cause of low compliance rate of some EBP may allow for more successful implementation of EBP interventions in the future. After further study, the SCC should be considered for scale up.

## Abbreviations

EBP: Essential birth practices
MDG: Millennium development goal
SCC: Safe childbirth checklist
SDG: Sustainable development goals
SPSS: Statistical package for the social sciences
WHO: World Health Organization
